# Concomitant boost IMRT-based neoadjuvant chemoradiotherapy for clinical stage II/III rectal adenocarcinoma: results of a phase II study

**DOI:** 10.1186/1748-717X-9-70

**Published:** 2014-03-07

**Authors:** Ji Zhu, Fangqi Liu, Weilie Gu, Peng Lian, Weiqi Sheng, Junyan Xu, Gang Cai, Debing Shi, Sanjun Cai, Zhen Zhang

**Affiliations:** 1 Department of Radiation Oncology, Fudan University Shanghai Cancer Center, No. 270, Dong’An Road, Shanghai 200032, China; 2 Department of Colorectal Surgery, Fudan University Shanghai Cancer Center, No. 270, Dong’An Road, Shanghai 200032, China; 3 Department of Pathology, Fudan University Shanghai Cancer Center, Shanghai 200032, China; 4 Department of Nuclear Medicine, Fudan University Shanghai Cancer Center, Shanghai 200032, China; 5 Department of Oncology, Shanghai Medical College, Fudan University, Shanghai 200032, China

**Keywords:** Rectal cancer, Intensity-modulated radiation therapy, Concomitant boost, Neoadjuvant chemoradiotherapy

## Abstract

**Aim:**

This study was designed to evaluate the efficacy and toxicities of concomitant boost intensity-modulated radiation therapy (IMRT) along with capecitabine and oxaliplatin, followed by a cycle of Xelox, in neoadjuvant course for locally advanced rectal cancer.

**Materials and methods:**

Patients with histologically confirmed, newly diagnosed, locally advanced rectal adenocarcinoma (cT3-T4 and/or cN+) located within 12 cm of the anal verge were included in this study. Patients received IMRT to the pelvis of 50 Gy and a concomitant boost of 5 Gy to the primary tumor in 25 fractions, and concurrent with oxaliplatin (50 mg/m^2^ d1 weekly) and capecitabine (625 mg/m^2^ bid d1–5 weekly). One cycle of Xelox (oxaliplatin 130 mg/m^2^ on d1 and capecitabine 1000 mg/m^2^ twice daily d1–14) was given two weeks after the completion of chemoradiation, and radical surgery was scheduled eight weeks after chemoradiation. Tumor response was evaluated by tumor regression grade (TRG) system and acute toxicities were evaluated by NCI-CTC 3.0 criteria. Survival curves were estimated using the Kaplan-Meier method and compared with Log-rank test.

**Results:**

A total of 78 patients were included between March 2009 and May 2011 (median age 54 years; 62 male). Seventy-six patients underwent surgical resection. Twenty-eight patients underwent sphincter-sparing lower anterior resection and 18 patients (23.7%) were evaluated as pathological complete response (pCR). The incidences of grade 3 hematologic toxicity, diarrhea, and radiation dermatitis were 3.8%, 10.3%, and 17.9%, respectively. The three-year LR (local recurrence), DFS (disease-free survival) and OS (overall survival) rates were 14.6%, 63.8% and 77.4%, respectively. Initial clinical T stage and tumor regression were independent prognostic factors to DFS.

**Conclusion:**

An intensified regimen of concomitant boost radiotherapy plus concurrent capecitabine and oxaliplatin, followed by one cycle of Xelox, can be safely administered in patients with locally advanced rectal cancer, and produces a high rate of pCR. A prognostic score model is helpful to distinguish different long-term prognosis groups in early stage.

## Introduction

Preoperative chemoradiotherapy (CRT) followed by total mesorectal excision (TME) is the standard treatment for patients with locally advanced rectal cancer (LARC). Some significant benefits of neoadjuvant CRT, such as better local control and sphincter preservation, have been shown in patients with stage II/III rectal cancer [1-3].

To obtain a better tumor response, elevating treatment dose has been considered a feasible method. EORTC22921 and FFCD9203 studies showed that preoperative radiotherapy combined with fluorouracil (5-FU) can significantly improve the treatment effect compared with radiotherapy alone [3,4]. In a retrospective analysis of 3,157 patients enrolled in seven randomized Phase III trials and 45 Phase II trials, the use of continuous infusion 5-FU, a second drug based on 5-FU and a higher radiation dose was associated with higher rates of pCR [5]. However, the next five randomized phase III trials, ACCORD 12/0405-Prodige 2 [6], STAR-01 [7], NSABP R-04 [8], CAO/ARO/AIO-04 [9] and PETACC-6 [10], demonstrated conflicting results as to whether oxaliplation increased the rate of pCR.

In our center, most patients receiving neoadjuvant CRT were clinical T4 or N+, and might have more opportunities to benefit from a high intensity treatment, whether chemotherapy or radiotherapy [11-13]. To decrease the additional toxicities from a high-dose treatment, especially diarrhea, intensity modulated radiotherapy (IMRT) was used to lessen radiation-associated toxicities by decreasing the volume of high irradiation dose of surrounding normal tissues [14-16]. IMRT allows higher radiation doses to be focused on regions within the tumor while minimizing the dose to surrounding normal critical structures. The data from dosimetric studies of IMRT in rectal cancer are encouraging. Compared to conventional 2D or 3D radiation therapy, IMRT showed similar target coverage with reduced dose to the small bowel, bladder, pelvic bone and femoral heads [14,17,18]. By decreasing the dose delivered to normal structures, IMRT may provide a potential for increasing treatment dose to improve tumor response.

Therefore, we designed this study to examine the use of IMRT, escalating the primary lesion’s dose to 55 Gy together with the whole pelvis dose of 50 Gy in 25 fractions, along with weekly capecitabine and oxaliplatin. Two weeks after the end of chemoradiotherapy, a cycle of Xelox (capecitabine and oxaliplatin) was prescribed before surgery. The efficacy and toxicity of this modality were evaluated to explore the feasibility of high-dose intensity in preoperative treatment. This phase II study was approved by our institutional review board.

## Materials and methods

### Eligibility criteria

Patients with histologically confirmed, newly diagnosed, locally advanced rectal adenocarcinoma (cT3-T4 and/or cN+) located within 12 cm from the anal verge were included in this study at the Fudan University Shanghai Cancer Center. All patients were ≥ 18 years of age and had a Karnofsky Performance Status score of ≥ 60, no evidence of distant metastases, adequate bone marrow function (leukocyte count > 4,000/mL and platelet count > 100,000/mL), and adequate renal and hepatic function (creatinine clearance > 50 mL/min and bilirubin ≤ 2 mg/mL). Patients were excluded if they were older than 75 years of age, had undergone previous pelvic radiotherapy or previous chemotherapy, or had previous or synchronous tumors other than nonmelanoma skin cancer. Patients suffering of the following medical conditions were also ineligible: ischemic heart disease, inflammatory bowel disease, malabsorption syndrome, peripheral neuropathy, or psychological disorders. Signed informed consent was obtained from all patients before inclusion on this study. The institutional review board of Fudan University approved the study.

### Baseline evaluation

Pretreatment evaluation was performed within two weeks before initiation of chemoradiation. The evaluation included a complete history and physical examination, including digital rectal examination, complete blood count, hepatic and renal function tests, tumor marker measurement, colonoscopy and biopsy, computed tomography (CT) of the thorax and abdomen, magnetic resonance imaging (MRI) of the pelvis, and, in selected patients, endorectal ultrasound. All patients were clinically staged with the AJCC 7th version manual.

### Combined chemoradiotherapy

#### Intensity modulated radiation therapy (IMRT)

All patients were immobilized in the prone position using a belly board and underwent a non-contrast-enhanced, planning CT with 5-mm slices from the L3-L4 junction to 2 cm below the perineum. The image data were transferred to the PINNACLE planning system (Philips Radiation Oncology Systems, Milpitas, CA). The definitions of volumes were in accordance with the ICRU Report #83 [19]. The gross tumor volume (GTV) was defined as all known gross disease determined from CT and MRI. The clinical target volume 1 (CTV1) included the gross tumor volume and the corresponding mesorectum plus 2 cm cranio-caudally. The CTV2 included the CTV1 plus the entire mesorectum, entire pre-sacral space, internal iliac nodes and high-risk anatomical and nodal sub-sites, based on the distance of the tumor from the anal margin [20]. Based on our institution set-up data, the planning target volume (PTV) was defined as the CTV with 10-mm margins superiorly and inferiorly and 8-mm margins in all other directions. Organs at risk (OARs) were contoured as follows: 1) the small intestine was defined as all intestinal loops below the sacral promontory (rectosigmoid junction excluded); 2) femoral heads were contoured from the cranial extremity to the level of the lower margin of ischial tuberosities; and 3) the bladder was contoured entirely with no distinction between the wall and its content [16]. The IMRT plans were generated using the inverse planning module of PINNACLE for a 6-MV liner accelerator, with five to seven coplanar fields.

The planned doses to the PTV1 and PTV2 were 55 Gy and 50 Gy, respectively, in 25 fractions, five times per week (Monday through Friday) for five weeks. The D2%, D50%, and D98% to PTV1 and PTV2 were set at 52.25 Gy and 55 Gy, 57.75 Gy and 47.5 Gy, and 50 Gy and 52.5 Gy, respectively. The doses of the OARs were set as low as possible and had to at least meet the following constraints: bladder, ≥ 45 Gy in 15% volume and ≥ 40 Gy in 40% volume; femoral heads, ≥ 45 Gy in 25% volume and ≥ 40 Gy in 40% volume; and small bowel, ≥ 45 Gy in 65 cc volume, ≥ 40 Gy in 100 cc volume, and ≥ 35 Gy in 180 cc volume.

The positioning and isocenter of each patient were verified on electronic portal imaging device (EPID) films for the anterior and lateral gantry positions by visually comparing the digitally reconstructed radiographs.

#### Concurrent and neoadjuvant chemotherapy

**Figure 1:**
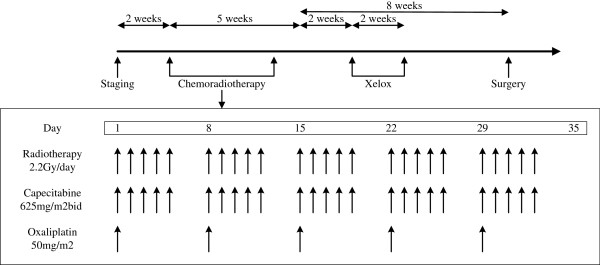
The workflow of preoperative chemoradiotherapy in patients with locally advanced rectal cancer.

Capecitabine combined with oxaliplatin was administered concurrently with pelvic radiation. Capecitabine was given at a dose of 625 mg/m^2^ twice daily from Monday to Friday throughout the entire course of IMRT. Oxaliplatin at a dose of 50 mg/m^2^ was administered weekly during the five-week course of radiotherapy. Two weeks after concurrent chemoradiation, one cycle of Xelox (oxaliplatin 130 mg/m^2^ on d1 and capecitabine 1000 mg/m^2^ twice daily d1–14) was administered (Figure 1).

#### Surgery and histopathology

Surgery was scheduled eight weeks after the completion of CRT. Total mesorectal excision (TME) was mandatory, whereas the form of surgery (anterior resection or abdominal-perineal resection) and whether a temporary colostomy should be performed were decided by the surgeon. All lymph nodes were examined according to standard procedures. If the number of lymph nodes was less than 12, two pathologists were needed to sign to ensure the reliability of the detection result. The circumferential rectal margin (CRM) was assessed according to the method of Quirke et al. [2], and a margin of < 1 mm was considered CRM-positive. All sections of the surgical specimens were reviewed by two pathologists. The pathologic stage (ypTN) was recorded according to the Union for International Cancer Control (UICC) TNM system. Tumor regression grading (TRG) was evaluated according to the criteria by Dworak et al. as follows [21]: Grade 0, no regression; Grade 1, dominant tumor mass with obvious fibrosis and/or vasculopathy; Grade 2, dominant fibrotic changes with few tumor cells or groups (easy to find); Grade 3, very few (difficult to find microscopically) tumor cells in fibrotic tissue with or without mucous substance; and Grade 4, no tumor cells, only a fibrotic mass (total regression or response).

#### Adjuvant chemotherapy and follow-up

All patients were recommended to receive postoperative chemotherapy regardless of pathological stages. Adjuvant chemotherapy was recommended consisting of five cycles of Xelox. Patient follow-up was scheduled every three months during the first two years, and then every six months over the next three years. After five years, the frequency of follow-up was extended to once each year.

#### Toxicity and measurement

Toxicities were evaluated and recorded weekly according to the CTC 3.0 criteria. If grade 3 toxicities occurred, the physicians determined causes and decided the response. In general, the sequence of dose reduction or suspension moved from oxaliplatin to capecitabine to radiotherapy, unless an adverse effect was strongly associated with a particular treatment.

#### Endpoints and statistics

The primary endpoint for this trial was pCR rate. This study was a phase II trial of 78 patients to evaluate the treatment feasibility and efficacy of this dosing regimen. Based on a literature review, the pCR rate is approximately 10–15% for patients treated with neoadjuvant CRT. We determined that an experimental arm with a pCR rate of at least 18% would merit further study. In this study, if more than 17 cases were evaluated as pCR, we had 85% power to reject the null hypothesis that our strategy could not reach the pCR of 18%, with a type I error level of 5%. Secondary endpoints included safety, sphincter preservation rate, TRG, LR (local recurrence), DFS (disease-free survival) and OS (overall survival). Sphincter preservation was defined as any procedure in which the rectal tumor was removed while leaving behind the anal sphincter.

All characteristics were described by the frequency for classified variables, by mean and standard deviations for normal distributional continuous data, and by the median for non-normal distributional continuous data.

Survival time was calculated from the beginning of CRT to the date of event or the last follow-up. Survival curves were estimated using the Kaplan-Meier method and compared with Log-rank test. Cox proportional hazards regression was used for multivariate modeling and for examining the prognostic significance of the variables identified in the models. P values of less than 0.05 were taken to indicate statistically significant differences.

## Results

### Clinical characteristics

Between March 2009 and May 2011, a total of 78 patients were included in the study. All patients were diagnosed with locally advanced rectal cancer: 50 with cT3 and 28 with cT4 primary tumor. Lymph node involvements were detected in 75 patients. Of the total 78 patients, 62 were men and 16 were women; the median age was 54 years (range, 30–76 years). Fifty-six patients (71.8%) had tumors located ≤ 5 cm from the anal verge (Table 1).

**Table 1:** Demographic and clinical features for all patients

	**n**	**%**
Gender		
Male	62	79.5
Female	16	20.5
Age, years		
Median (min-max)	54(30–76)	
Distance from anal verge		
≤ 5 cm	56	71.8
> 5 cm	22	28.2
cT stage		
T3	50	64.1
T4	28	35.9
cN stage		
N0	3	3.8
N1	34	43.6
N2	41	52.6
Total	78	100.0

### Treatment compliance and acute toxicities

All patients completed the prescribed radiation treatment to a total dose of 55 Gy in 25 fractions. The median total radiation duration was 37 days (range, 33–41). All patients completed five weeks of capecitabine, and 48 cases received five cycles oxaliplatin and the rest received four cycles. In addition, all patients received a scheduled single cycle of Xelox two weeks after the completion of chemoradiotherapy without dose adjustment.

Most of the adverse events during CRT were mild (grade 1 or 2). No grade 4–5 toxicities were observed. The most common grade 3 toxicity was radiation dermatitis (17.9%), while grade 3 diarrhea and hematological toxicities were evaluated in eight (10.3%) and three cases (3.8%) (Table 2).

**Table 2:** Toxicity during the course of chemoradiation

	**Grade 1**		**Grade 2**		**Grade 3**	
	**n**	**%**	**n**	**%**	**n**	**%**
Diarrhea	15	19.2%	11	14.1%	8	10.3%
Hematologic	13	16.7%	3	3.8%	3	3.8%
Fatigue	10	12.8%	5	6.4%	3	3.8%
Radiation dermatitis	20	25.6%	16	20.5%	14	17.9%
Neurosensory	3	3.8%	1	1.3%	0	0.0%
Hand-foot syndrome	0	0.0%	1	1.3%	0	0.0%

### Surgical procedures and pathological response

Seventy-six patients underwent a surgical resection according to the schedule. One patient refused surgery because of good response, and another case did not receive an operation because of being evaluated as unresectable lesions. The median interval between the completion of CRT and primary tumor surgery was 52 days (range, 46–67 days). Twenty-eight patients (36.8%) underwent sphincter-sparing lower anterior resection. All pathological features are listed in Table 3. ypT0 and ypN0 were found in 18 (23.7%) and 47 (61.8%) patients, respectively, with 18 patients (23.7%) showing pCR. TRG information was available in pathologic examination for all 76 patients receiving surgery. The TRG stage was Grade 4 (pCR) in 18 patients, Grade 3 in 34 patients, Grade 2 in 17 patients, and Grade 1 in 7 patients. All cases were divided into two subgroups: good responders (defined as TRG 3–4) or poor responders (defined as TRG 1–2). More than or equal to 12 lymph nodes were found in half of the patients. Lymphatic/vascular invasion and neural invasion were confirmed in five and eight cases, respectively (Table 3). The overall rate of postoperative complications was 17.1%. Delayed sacral-wound healing, postoperative bleeding and anastomotic leakage occurred in 9, 3 and 1 patients, respectively.

**Table 3:** Surgical procedure and pathological findings

	**n**	**%**
Surgery		
Anterior resection	28	36.8%
Abdominal perineal resection	46	60.5%
Hartmann	2	2.6%
Lymphatic or vascular invasion		
No	71	93.4%
Yes	5	6.6%
Neural invasion		
No	68	89.5%
Yes	8	10.5%
CRM		
Negative	76	100.0%
Positive	0	0.0%
ypT stage		
T0	18	23.7%
T1	10	13.2%
T2	20	26.3%
T3	26	34.2%
T4	2	2.6%
ypN stage		
N0	47	61.8%
N1	19	25.0%
N2	10	13.2%
Examined lymph nodes		
Median (Min-max)	12(2–35)	
TRG Score		
4	18	23.1%
3	34	43.6%
2	17	21.8%
1	7	9.0%
Total	76	100.0%

CRM: circumferential resection margin.

TRG: tumor regression grade.

#### Follow-up

**Figure 2:**
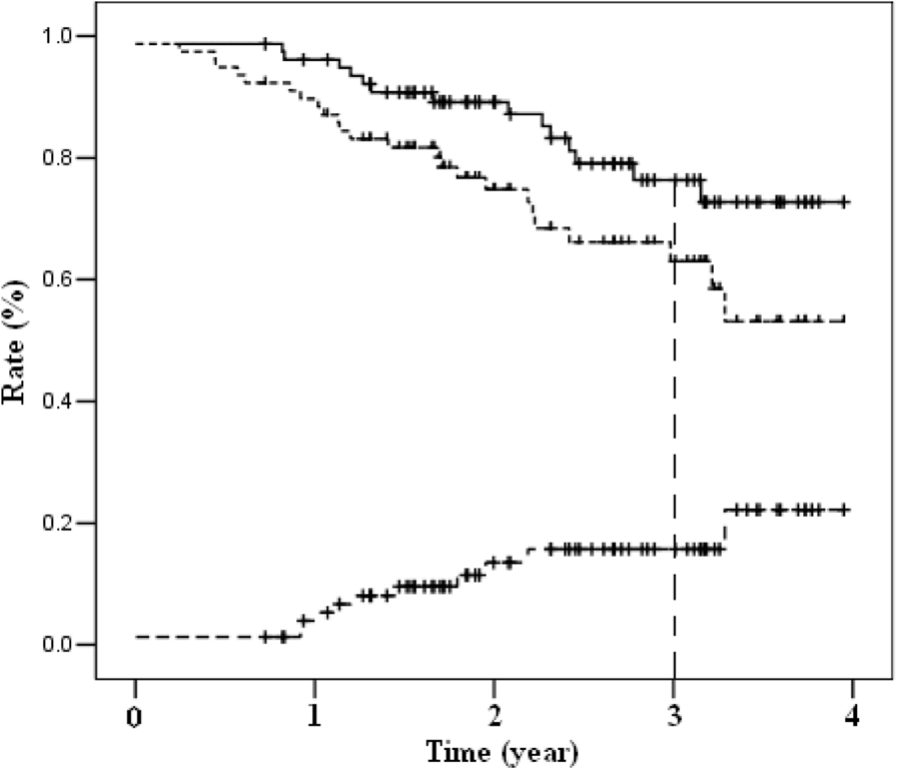
Survival curves of local recurrence, overall survival (OS) and disease-free survival (DFS).

With a median follow-up of 30 months (range, 9–48 months), 10 patients were diagnosed with local recurrence and 19 patients were confirmed with distant metastases (5 in the liver, 13 in the lung, and 1 in bone). Fourteen patients died of rectal cancer. For the two patients that did not receive surgery, one patient was confirmed of tumor failure at 27 months and died 28 months after the beginning of CRT, and the other patient did not present any evidence of failure at the last visit of 9 months. The 3-year LR, DFS and OS rates were 14.6%, 63.8% and 77.4%, respectively (Figure 2).

#### Univariate and multivariate analysis for LR and DFS

**Figure 3:**
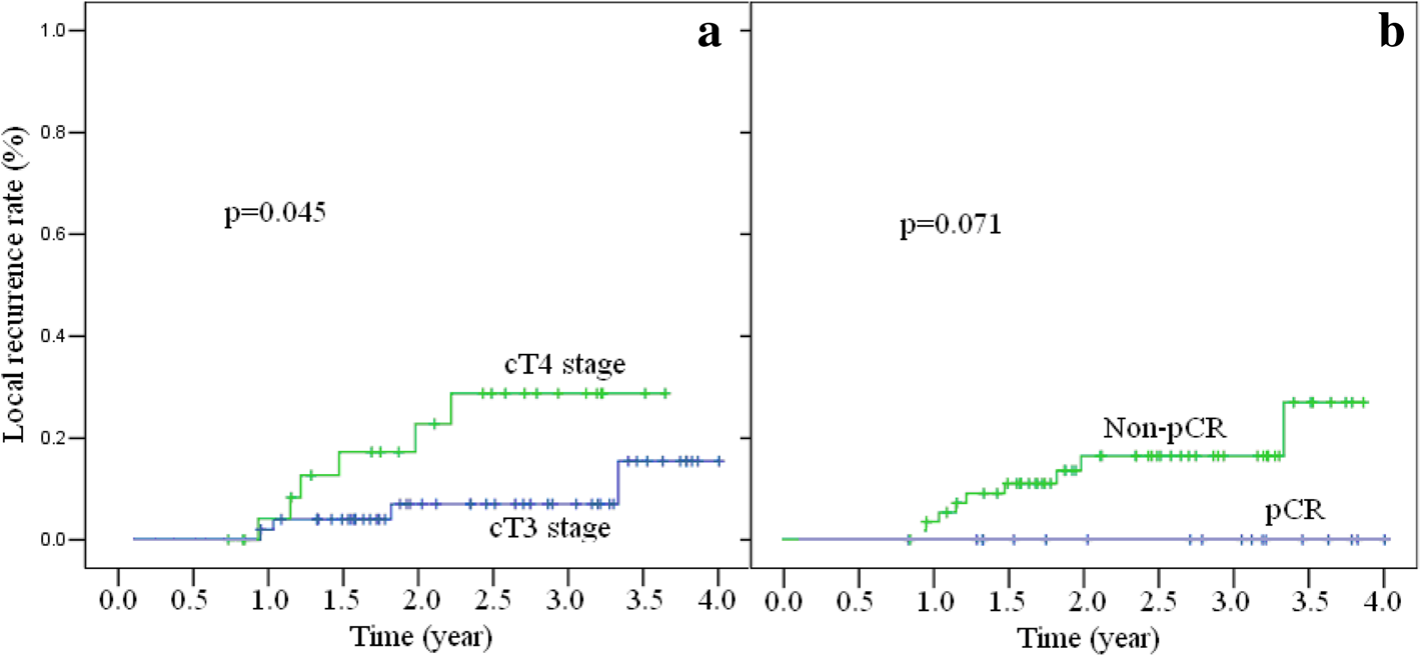
**Local recurrence rates by clinical T stage (panels a) and pCR or not (panels b).** pCR: pathological complete response.

All potential prognostic factors, including age, gender, distance from anal verge, cT stage, cN stage, ypT stage, ypN stage and TRG score were evaluated using the Kaplan-Meier method (compared with Log-rank test). cT stage and pCR status demonstrated a correlation with LR (Figure 3). No LR was observed in pCR cases. In the next multivariate Cox regression analysis, only cT stage was left in the model.

**Figure 4:**
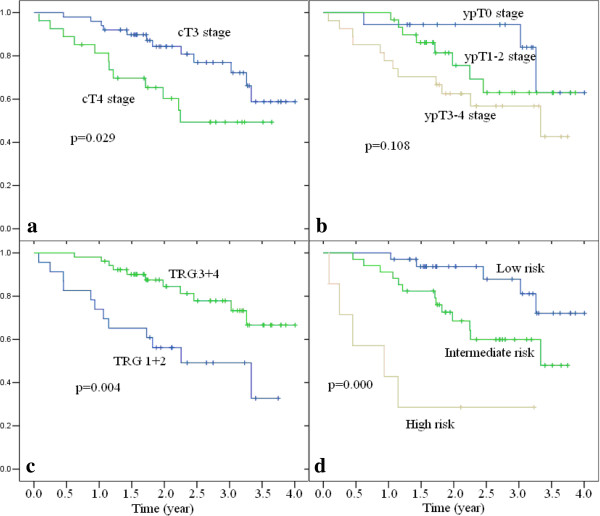
**Disease-free survival rates by clinical T stage (panels a), ypT stage (panels b), tumor regression (panels c) and prognostic score (panels d).** TRG: tumor regression grade.

cT stage, ypT stage and TRG score exhibit a correlation with DFS. YpT stage was excluded in the further multivariate Cox regression analysis. Hazard ratios of these two factors (cT stage and TRG score) were very close to one another (Table 4). A prognostic scoring system was produced, with each of the two unfavorable prognostic factors allocated one point, including cT4 stage and poor responder. Use of the scoring system led to the identification of three risk groups: low risk (score of 0), intermediate risk (score of 1), and high risk (score of 2). The three-year DFS rates were significantly different among the groups: the low-risk group, 81.1%; intermediate-risk group, 60.0%; and the high-risk group, 28.6% (*P* = 0.000, Figure 4).

**Table 4:** **The values of β, hazard ratio and *P *** **values in Cox multivariate regression model disease-free survival**

		**β**	**Hazard ratio**	***P*** **value**
LR	Clinical T stage (3 vs. 4)	1.23	3.43	0.05
DFS	TRG score (3/4 vs. 1/2)	1.36	3.90	0.00
	Clinical T stage (3 vs. 4)	1.11	3.04	0.01

LR: local recurrence.

DFS: disease-free survival.

TRG: tumor regression grade.

## Discussion

Our study findings demonstrated that the IMRT technique, which decreases radiation-induced toxicities by a lower high-dose irradiation volume, and an intensified preoperative CRT followed by a cycle of Xelox resulted in a pCR rate of 24.7%. These results are higher than those of studies on preoperative conventional CRT concurrent with oxaliplatin (15–20%) [6-10]. Regarding toxicities, however, the high rate of pCR did not translate into high local control compared to other trials. This may be attributed to two factors, First, our study had a small sample size, and therefore some certain remains. Second, the percentage of cT4 and cN + tumors in our study was significantly higher than some other trials (cT4: 35.9% vs. 5-15%, cN+: 96.2% vs. 40-70%, respectively) [1,3,5,7-11]. Our study found that the incidence of grade 3 diarrhea, hematologic toxicity, and radiation dermatitis was 10.3%, 3.8%, and 17.9%, respectively. Consistent with our previous study [22], the incidences of diarrhea and hematologic toxicity were slightly lower compared with other reported stage III clinical trials. A significant increase in the incidence of radiation dermatitis was observed in our study, which might be attributed to a lower irradiation field due to a distal rectal tumor location in most cases. Finally, with a median follow-up of 30 months, our data showed that the baseline T stage and treatment response were associated with long-term prognosis. Patients with cT4 stage had a higher chance of LR and distance metastases, but no LR was observed in pCR cases, regardless of initial T stage. In addition, baseline T stage and tumor response had equal effects to predict DFS. Patients with cT3 and good response had a three-year DFS of 81.1%, but for those with cT4 and poor response, the three-year DFS declined to 28.6%.

The IMRT technique has been used widely in some solid tumors. In our study, IMRT was used to concomitantly boost the total irradiation dose to 55 Gy for the gross tumor. The rationale was based on several dosimetric studies that showed that IMRT could significantly decrease the surrounding organs’ high-dose irradiation volume, especially the small bowel, compared with conventional radiotherapy or 3DCRT [14,15]. Therefore, IMRT provided the potential of elevating the dose to improve the tumor response. This is consistent with results from Hartley’s meta-analysis, which included a total of 3157 rectal cancer patients [5]. Our previous study of stage IV rectal cancer demonstrated the feasibility to deliver 45 Gy to the pelvis and a concomitant 10 Gy boost to the gross tumor using IMRT [23].

Oxaliplatin is an effective drug for colorectal cancer when combined with 5-FU. However, the results of five recent large sample phase III trials are unclear as to whether oxaliplatin is an appropriate radiation sensitizer. Three studies, including NSABP R-04 [8], STAR-01 [7] and PETACC-6 [10], all reported that additional oxaliplation based on conventional CRT failed to increase pCR rate and caused more toxicities, especially diarrhea. The ACCORD 12/0405-Prodige 2 trial also reported a higher toxicity rate in the oxaliplatin group, together with a significantly higher pCR rate [6]. The CAO/ARO/AIO-04 trial was the only phase III trial supporting additional oxaliplatin in preoperative CRT, which showed a better tumor regression in the oxaliplatin group without any additional toxicities [9]. However, our study demonstrated an encouraging pCR and tumor shrinkage, without high incidence of toxicities. This also illustrated that IMRT was an effective method to offset toxicities induced by high-dose CRT.

In several previous studies, early surrogate indicator was focused to help to decide the strategy of adjuvant therapy. Tumor regression after CRT, especially pCR, was regarded as an important prognostic factor. The EORTC 22921 trial showed that patients with good response had a significantly good prognosis compared with those with poor response [24]. Capirci’s retrospective study of 566 patients with pCR also demonstrated an encouraging prognosis [25]. In a retrospective study by MD Anderson Cancer Center, tumor response was associated with the five-year recurrence free survival, distant-metastasis rate and LR [26]. Thus, after neoadjuvant therapy, conventional adjuvant chemotherapy (oxaliplatin plus fluorouracil) might be over-treatment to patients with good prognosis, but no use to poor responders. Based on our data, a prognostic score model including initial clinical T stage and tumor response might be helpful to determine the adjuvant chemotherapy regimen.

## Conclusion

An intensified regimen of concomitant boost radiotherapy plus concurrent capecitabine and oxaliplatin, followed by one cycle of Xelox, can be safely administered in patients with locally advanced rectal cancer, and produces a high rate of pCR. A prognostic score model is helpful to distinguish different long-term prognosis groups in early stage.

## Competing interests

The authors declare that they have no competing interests.

## Authors’ contributions

ZJ and LFQ conceived and drafted the manuscript, ZZ and CSJ drafted and revised the manuscript, and all authors read and approved the final manuscript.
